# Supplementary material for: Food Sources and Dietary Quality in Small Island Developing States: Development of Methods and Policy Relevant Novel Survey Data from the Pacific and Caribbean
Source: Nutrients. 2020 Oct 30;12(11):3350. doi: 10.3390/nu12113350 (PMC7692177; doi:10.3390/nu12113350)
Supplement: Supplementary file 1 [file nutrients-12-03350-s001.pdf]

Supplementary Materials

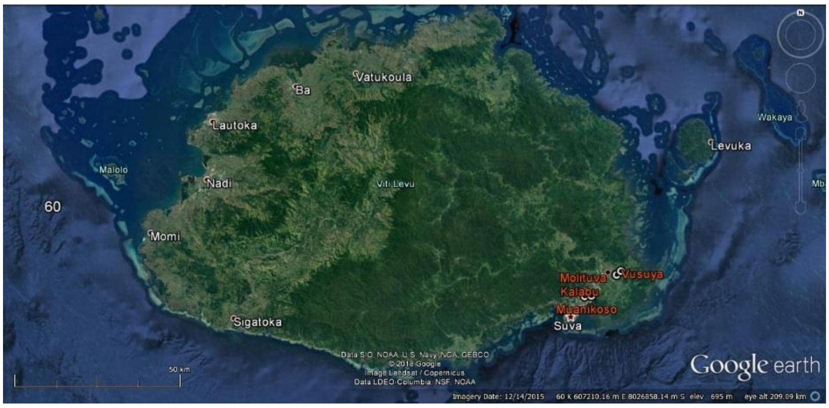

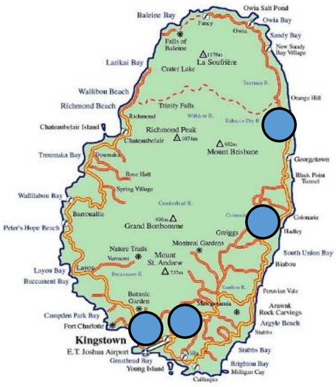

Figure 1. Islands of Fiji (top) and St Vincent (bottom), showing the approximate locations of the study sites in red writing and blue dots, respectively. Not to scale.

Table 1. Selected demographic and social characteristics of respondents in the CFaH project by ever sourcing food from own production, purchasing, borrowing/exchanging and food aid. *.

Food Source	Number (Col %)	Age (Mean (SD))	% Female	% > Primary Education	% Household Size >3	% Rural Residents
				FIJI		
Own production						
No	32 (17.3)	37.8 (16.0)	56.3	62.5	64.5	31.0
Yes	153 (82.7)	42.2 (17.2)	64.1	54.6	47.1	45.6
Diff (95% CIs)		4.4 (−2.1, 11.0)	7.8 (−11.0, 26.6)	−7.9 (−26.4, 10.7)	−17.5 (−36.1, 1.2)	14.6 (−4.0, 33.2)
Purchase						
No	5 (2.7)	40.6 (26.8)	60.0	40.0	60.0	60.0
Yes	181 (97.3)	41.4 (16.8)	63.0	56.1	50.0	43.1
Diff (95% CIs)		0.8 (−14.5, 16.1)	3.0[a]	16.1[a]	10.0[a]	−16.9[a]
Borrow/exchange						
No	143 (76.9)	41.1 (17.1)	62.9	57.0	51.4	40.4
Yes	43 (23.1)	42.2 (17.0)	62.8	51.2	46.5	53.5

Diff (95% CIs)		1.1 (−4.8, 7.0)	−0.1 (−16.6,16.3)	−5.9 (−22.9, 11.1)	−4.9 (−21.9, 12.1)	13.0 (−4.0, 30.1)
Food aid						
No	177 (95.2)	40.6 (16.7)	61.6	56.8	51.1	43.5
Yes	9 (4.8)	55.3 (18.4)	88.9	33.3	33.3	44.4
Diff (95% CIs)		14.7 (3.4, 26.0)	27.3 [a]	−23.5 [a]	17.8 [a]	0.9 [a]
				SVG		
Own production						
No	65 (44.2)	37.2 (17.7)	66.7	54.7	50.0	60.0
Yes	82 (55.8)	44.4 (18.3)	60.0	40.0	52.4	80.5
Diff (95% CIs)		7.2 (1.2, 13.1)	−6.7 (−22.5, 9.2)	−14.7 (−30.9, 1.6)	2.4 (−13.9, 18.8)	20.5 (5.8, 35.2)
Purchase						
No	10 (6.8)	47.9 (21.5)	40.0	25.0	60.0	70.0
Yes	137 (93.2)	40.7 (18.1)	64.7	47.8	50.7	71.5
Diff (95% CIs)		−7.2 (−19.0, 4.7)	24.7 (−6.8, 56.1)	22.8 (−8.4, 54.0)	−9.3 (−40.8, 22.2)	1.5 (−27.9, 30.9)
BEB [b]						
No	89 (60.5)	40.6 (18.5)	59.3	42.0	50.0	60.7
Yes	58 (39.5)	42.3 (18.3)	68.4	53.6	53.4	87.9
Diff (95% CIs)		1.7 (−4.5, 7.9)	9.1 (−6.8, 25.0)	11.5 (−5.1, 28.2)	3.4 (−13.1, 20.0)	27.3 (14.1, 40.4)
Food aid						
No	144 (98.0)	41.0 (18.3)	62.1	47.5	52.4	71.5
Yes	3 (2.0)	54.3 (15.5)	100	0	0	66.7
Diff (95% CIs)		13.4 (−7.7, 34.5)	37.9 [a]	−47.5 [a]	−52.4 [a]	−4.9 [a]

* Shaded cells indicate where the 95% CIs on the difference do not cross zero; [a] CIs not computed due to small numbers in one or more categories. All *p* values (Fisher's exact test) >0.1; [b] borrow, exchange or barter.

Table 2. Types of food borrowed/exchanged by those reporting sourcing food in this way more than once a week in the CFaH project.

	Fiji (n = 10)	SVG (n = 48)
	n (%)	n (%)
Roots/tubers/plantains	4 (40%)	39 (81%)
Vegetables	4 (40%)	16 (33%)
Fruit	2 (20%)	18 (38%)
Fish	4 (40%)	9 (19%)
Meat (unprocessed)	3 (30%)	0
Sweets	0	9 (19%)
SSBs *	0	8 (17%)

* Sugar-sweetened beverages.

Table 3. Associations between greater than weekly use of selected food sources in the CFaH project. Figures are odds ratios (95% confidence intervals). Figures for Fiji are in the bottom left hand side of the table, and for SVG in the top right hand side.

		SVG					
		**Own Prod**	**S-Market**	**F-Shop**	**Inf-Shop**	**Food Serv**	**BEB** [a]
**Fiji**	**Own Prod**		2.0 (0.9, 4.1)	0.8 (0.4, 1.6)	1.2 (0.6, 2.4)	3.1 (0.8, 12.4)	5.1 (2.4, 10.8)

**S-Market**	0.7 (0.3, 1.2)		1.1 (0.5, 2.3)	2.4 (1.0, 5.7)	1.6 (0.3, 8.1)	5.1 (1.9, 14.1)
**F-Shop**	0.7 (0.3, 1.7)	2.1 (0.8, 5.7)		2.2 (1.1, 4.4)	1.1 (0.3, 4.2)	1.0 (0.5, 2.0)
**Inf-Shop**	1.8 (0.8, 4.2)	3.5 (1.5, 8.6)	29.0 (9.6, 87.6)		1.5 (0.4, 5.6)	4.7 (2.2, 9.9)
**Food Serv**	0.8 (0.3, 2.6)	1.8 (0.5, 6.0)	14.0 (4.3, 45.6)	14.0 (4.1, 48.1)		0.5 (0.1, 2.4)
**BEB** [a]	0.7 (0.2, 2.5)	1.0 (0.0, 0.0)	3.3 (0.8, 14.0)	4.7 (1.3, 17.1)	1.4 (0.2, 11.9)	

* Shaded cells indicate where the 95% CIs on the odds ratio do not cross 1; [a] borrow, exchange or barter.

Table 4. Overweight or obesity and hypertension by socio-demographic characteristics, food sources and aspects of diet in the CFaH project in Fiji. *.

Variable	Overweight or obesity **			Hypertension ***		
	**Yes**	**No**	**Diff (95% CIs)**	**Yes**	**No**	**Diff (95% CI)**
Age (mean (SD))	45.0 (16.4)	32.3 (15.0)	12.7 (7.6, 17.8)	53.4 (14.6)	33.3 (13.9)	20.1 (15.8, 24.4)
Sex (% female)	72.3	40.0	32.3 (17.2, 47.4)	66.7	59.3	7.4 (-6.9, 21.7)
>Primary education (%)	54.3	60.0	−5.7 (−21.3, 9.8)	43.1	63.0	−19.9 (−34.5, −5.3)
Household size > 3 (%)	49.2	53.7	−4.5 (−20.3, 11.4)	43.1	53.3	−10.2 (−25.1, 4.6)
Region (% rural)	42.4	47.2	−4.8 (−20.8, 11.2)	34.3	48.5	−14.3 (−29.0, 0.5)
>Than weekly use of food source						
Own production	72.3	58.2	14.1 (−1.0, 29.3)	66.7	69.4	−2.8 (−16.7, 11.2)
Supermarket	59.2	58.2	1.0 (−14.5, 16.6)	61.1	57.4	3.7 (−10.9, 18.3)
Small shop	21.5	23.6	−2.1 (−15.4, 11.2)	23.6	22.2	1.4 (−11.2, 13.9)
Formal small shop	10.8	16.4	−5.6 (−16.7, 5.5)	11.1	13.9	−2.8 (−12.5, 7.0)
Informal small shop	20.0	18.2	1.8 (−10.5, 14.1)	22.2	18.5	3.7 (−8.4, 15.8)
Food service business	6.9	9.1	−2.2 (−10.9, 6.6)	6.9	8.3	−1.4 (−9.2, 6.5)
BEB [a]	5.4	5.5	−0.1 (−7.2, 7.1)	9.7	2.8	6.9 (−0.6, 14.5)
DDS [b] (mean (SD))	3.8 (1.4)	3.4 (1.5)	0.3 (−0.2, 0.8)	3.7 (1.5)	3.6 (1.4)	0.1 (−0.4, 0.6)
Median weekly servings						
Fruit	3	2	1 (0, 2)	2	2	0 (−1, 1)
Vegetables	7	7	0 (−3, 3)	7	7	0 (−3, 3)
SSB [c]	2	4	−2 (−5, 1)	2	3	−1 (−4, 2)
Processed or red meat	2	3	−1 (−2, 0)	2	2	0 (−1, 1)

* Shaded cells indicate where 95% CIs on the difference do not cross zero; ** defined as body mass index ≥25 kgm[2]; *** defined as on treatment and/or measured blood pressure of ≥140/90 mmHg; [a] borrow, exchange or barter; [b] the dietary diversity score; [c] sugar-sweetened beverages.

Table 5. Overweight or obesity and hypertension by socio-demographic characteristics, food sources and aspects of diet in the CFaH project in SVG. *.

Variable	Overweight or obesity **			Hypertension ***		
	**Yes**	**No**	**Diff (95% CIs)**	**Yes**	**No**	**Diff (95% CI)**
Age (mean (SD))	45.5(16.5)	32.6 (18.8)	12.9 (6.9, 18.9)	55.9 (15.2)	35.0 (16.3)	20.9 (14.9, 26.9)
Sex (% female)	75.3	40.8	34.5 (18.0, 50.9)	64.1	62.9	1.2 (-16.6, 19.1)
> Primary education (%)	42.2	56.9	−14.6 (−31.6, 2.4)	25.6	54.0	−28.4 (−45.2, −11.5)
Household size > 3 (%)	54.4	47.1	7.4 (−9.7, 24.5)	46.2	53.0	−6.8 (−25.3, 11.6)
Region (% rural)	69.2	72.5	−3.3 (−18.8, 12.2)	62.5	73.0	−10.5 (−27.8, 6.8)
>Than weekly use of food source						
Own production	46.2	41.2	5.0 (−12.0, 21.9)	42.5	45.0	−2.5 (−20.7, 15.7)
Supermarket	74.7	70.6	4.1 (−11.2, 19.5)	70.0	73.0	−3.0 (−19.7, 13.7)
Small shop	48.4	60.8	−12.4 (−29.3, 4.4)	47.5	55.0	−7.5 (−25.8, 10.8)
Formal small shop	33.0	43.1	−10.2 (−26.8, 6.5)	25.0	41.0	−16.0 (−32.5, 0.5)
Informal small shop	31.9	31.4	0.5 (−15.4, 16.4)	32.5	31.0	1.5 (−15.6, 18.6)
Food service business	5.5	9.8	−4.3 (−13.7, 5.1)	2.5	9.0	−6.5 (−13.9, 0.9)

BEB [a]	36.3	25.5	10.8 (−4.7, 26.3)	35.0	30.0	5.0 (−12.3, 22.3)
DDS [b] (mean (SD))	4.0 (1.6)	3.6 (1.4)	0.3 (−0.2, 0.9)	4.0 (1.9)	3.7 (1.4)	0.3 (−0.3, 0.9)
Median weekly servings						
Fruit	7	7	0 (−5, 5)	7	7	0 (−5, 5)
Vegetables	3	5	−2 (−4, 0)	4	4	0 (−3, 3)
SSB [c]	7	14	−7 (−13, −1)	3	12	−9 (−17, −3)
Processed or red meat	2	3	−1 (−3, 1)	3	2	1 (−1, 3)

* Shaded cells indicate where 95% CIs on the difference do not cross zero; ** defined as body mass index ≥25 kgm^2; *** defined as on treatment and/or measured blood pressure of ≥140/90 mmHg; [a] borrow, exchange or barter; [b] the dietary diversity score; [c] sugar-sweetened beverages.

Table 6. Results from logistic regression analyses in the CFaH project, with overweight or obesity and hypertension, respectively, as the dependent variable. All independent variables entered together; with adjustment for household sampling.

	**Odds Ratio (95% CIs)**	***p* Value**
Overweight or obesity *		
Age (years)	1.05 (1.03, 1.07)	0.000
Sex (female vs. male)	4.91 (2.69, 8.98)	0.000
Weekly SSB[a] servings	0.98 (0.94, 1.02)	0.249
Weekly fruit servings	1.01 (0.98, 1.05)	0.443
Weekly vegetable servings	0.99 (0.95, 1.04)	0.772
Weekly red or processed meat servings	0.97 (0.92, 1.02)	0.243
Country (SVG vs. Fiji)	0.77 (0.37, 1.61)	0.486
Hypertension **		
Age (years)	1.09 (1.06, 1.11)	0.000
Education	1.09 (0.50, 2.37)	0.819
Weekly SSB [a] servings	0.98 (0.95, 1.02)	0.319
Weekly fruit servings	1.00 (0.96, 1.05)	0.960
Country (SVG vs. Fiji)	0.52 (0.25, 1.08)	0.081

* defined as body mass index ≥25kgm^2. ** defined as on treatment and/or measured blood pressure of ≥140/90 mmHg; [a] sugar-sweetened beverages.
